# Phytochemicals as antibiotic alternatives to promote growth and enhance host health

**DOI:** 10.1186/s13567-018-0562-6

**Published:** 2018-07-31

**Authors:** Hyun Lillehoj, Yanhong Liu, Sergio Calsamiglia, Mariano E. Fernandez-Miyakawa, Fang Chi, Ron L. Cravens, Sungtaek Oh, Cyril G. Gay

**Affiliations:** 1 Animal Biosciences and Biotechnology Laboratory, Agricultural Research Service, US Department of Agriculture, Beltsville, MD 20705 USA; 2 University of California, Davis, CA 95616 USA; 3 Animal Nutrition and Welfare Service, Universitat Autònoma de Barcelona, 08193 Bellaterra, Spain; 4 Instituto de Patobiología, Centro Nacional de Investigaciones Agropecuarias, Instituto Nacional de Tecnología Agropecuaria, Calle Las Cabañas y Los Reseros s/n, Casilla de Correo 25, Castelar, 1712 Buenos Aires, Argentina; 5 Amlan International, Chicago, IL 60611 USA; 6 National Program Staff-Animal Health, Agricultural Research Service, US Department of Agriculture, Beltsville, MD 20705 USA

## Abstract

There are heightened concerns globally on emerging drug-resistant superbugs and the lack of new antibiotics for treating human and animal diseases. For the agricultural industry, there is an urgent need to develop strategies to replace antibiotics for food-producing animals, especially poultry and livestock. The 2^nd^ International Symposium on Alternatives to Antibiotics was held at the World Organization for Animal Health in Paris, France, December 12–15, 2016 to discuss recent scientific developments on strategic antibiotic-free management plans, to evaluate regional differences in policies regarding the reduction of antibiotics in animal agriculture and to develop antibiotic alternatives to combat the global increase in antibiotic resistance. More than 270 participants from academia, government research institutions, regulatory agencies, and private animal industries from >25 different countries came together to discuss recent research and promising novel technologies that could provide alternatives to antibiotics for use in animal health and production; assess challenges associated with their commercialization; and devise actionable strategies to facilitate the development of alternatives to antibiotic growth promoters (AGPs) without hampering animal production. The 3-day meeting consisted of four scientific sessions including vaccines, microbial products, phytochemicals, immune-related products, and innovative drugs, chemicals and enzymes, followed by the last session on regulation and funding. Each session was followed by an expert panel discussion that included industry representatives and session speakers. The session on phytochemicals included talks describing recent research achievements, with examples of successful agricultural use of various phytochemicals as antibiotic alternatives and their mode of action in major agricultural animals (poultry, swine and ruminants). Scientists from industry and academia and government research institutes shared their experience in developing and applying potential antibiotic-alternative phytochemicals commercially to reduce AGPs and to develop a sustainable animal production system in the absence of antibiotics.

## Introduction

Antibiotics, since their discovery in the 1920s, have played a critical role in contributing to the economic effectiveness of animal production as feed supplements at sub-therapeutic doses, to improve growth and feed conversion efficiency, and to prevent infections [1]. In-feed antibiotics (IFAs) are a common and well-established practice in the animal industry that has contributed to the intensification of modern day livestock production. However, with intensification of animal agriculture, concerns exist that the use of IFAs leads to development of antimicrobial resistance, posing a potential threat to human health [2]. Although mixed opinions still exist on the transfer of antibiotic resistance genes from animal pathogens to those of humans, studies have shown a potential link between the practice of using sub-therapeutic doses of antibiotics and the development of antimicrobial resistance among the microbiota.

In the US, antibiotic use in livestock and poultry feeds is under scrutiny as a result of increasing consumer awareness and the demand for livestock products from antibiotic-free production systems. In 2013, the US Food and Drug Administration (FDA) called for major manufacturers of medically important animal drugs to voluntarily stop labeling them for animal growth promotion [3], and published its final rule of the Veterinary Feed Directive (VFD) in 2015. The quest for alternative products has clearly intensified in recent years with the increase in regulations regarding the use of antibiotic growth promoters (AGPs) and the rise in consumer demand for poultry products from “Raised Without Antibiotics” or “No Antibiotics Ever” flocks [2].

There has been a significant increase in scientific papers in the recent literature on antibiotic alternatives and feed additives to promote growth and enhance gut health, and reduce the use of antibiotics in animal production. The classes of antibiotic alternatives that are available to increase animal productivity and help poultry and pigs perform to their genetic potential under existing commercial conditions include probiotics, organic acids, phytogenics, prebiotics, synbiotics, enzymes, antimicrobial peptides, hyperimmune egg antibodies, bacteriophages, clay and metals [2]. Although the beneficial effects of many of the alternatives developed have been well demonstrated, there is a lack of information on their mechanism of action, efficacy, and advantages and disadvantages of their applications in the field. Furthermore, the general consensus is that these products lack consistency and their efficacies vary among farms and locations. Therefore, their modes of action need to be better defined. Optimal combinations of various alternatives coupled with good management and husbandry practices will be the key to maximize performance and maintain animal productivity while we move forward, with the ultimate goal of reducing antibiotic use in the animal industry.

With declining AGPs usage and increasing consumers’ concerns about superbugs, the quest for novel alternate replacements to mitigate antibiotic use in animal agriculture will grow significantly in the coming years. In this Phytochemical Session, we reviewed scientific evidence that phytochemicals stimulate innate immune cells, reduce oxidative stress, maintain gut integrity, promote beneficial bacteria growth, and reduce the negative consequences of inflammation caused by enteric infections as effective antibiotic alternatives to promote animal growth performance in poultry, swine, and beef and dairy production.

## Plant-derived phytochemicals as antibiotic alternatives

Phytochemicals, also referred to as phytobiotics or phytogenics, are natural bioactive compounds that are derived from plants and incorporated into animal feed to enhance productivity [2]. Ideal antibiotic alternatives should have the same beneficial effects of AGPs, ensure optimum animal performance, and increase nutrient availability. Considering the proposed mechanism of action of AGPs in modulating the gut microbiome and immunity, a practical alternative should exert a positive impact on feed conversion and/or growth [2, 4]. Phytochemicals can be used in solid, dried and ground form or as extracts (crude or concentrated), and also can be classified as essential oils (EOs; volatile lipophilic substances obtained by cold extraction or steam/alcohol distillation) and oleoresins (extracts derived by non-aqueous solvents) depending on the process used to derive the active ingredients [2]. The main bioactive compounds of the phytochemicals are polyphenols, and their composition and concentration vary according to the plant, parts of the plant, geographical origin, harvesting season, environmental factors, storage conditions, and processing techniques [2].

In recent years, phytochemicals have been used as natural growth promoters in the ruminants, swine and poultry industries. A wide variety of herbs and spices (e.g., thyme, oregano, rosemary, marjoram, yarrow, garlic, ginger, green tea, black cumin, coriander and cinnamon) have been used in poultry for their potential application as AGP alternatives [2]. In contrast, several other phytochemicals such as grape pomace, cranberry fruit extract, *Macleaya cordata* extract, garlic powder, grape seed extract, and yucca extract, when tested as growth promoters, did not show any effects on performance parameters [2]. In addition to herbs and spices, various EOs (thymol, carvacrol, cinnamaldehyde, and eugenol, coriander, star anise, ginger, garlic, rosemary, turmeric, basil, caraway, lemon and sage) have been used individually or as blends to improve animal health and performance [2]. Variable results have been reported with the use of EOs in poultry diets, some including cinnamaldehyde [5–7], and a blend of thymol and cinnamaldehyde improved body weight gain in broilers, while others like thymol and EOs from star anise improved feed efficiency, as seen by reduced feed conversion ratio (FCR). Curcuma alone or curcuma with capsicum [7, 8] enhanced resistance to enteric diseases such as coccidiosis and necrotic enteritis. The variation in the results could be attributed to differences in the composition, type and origin of the EOs that were used, inclusion level, and the environmental conditions of the trials [2]. Nevertheless, one commercial blend of phytonutrients (containing carvacrol, cinnamaldehyde and *Capsicum* oleoresin), which enhances innate immunity and reduces negative effects of enteric pathogens [9, 10], was approved in the EU as the first botanical feed additive for improving performance in broilers and livestock. Several trials performed with this commercial blend have demonstrated consistent improvement in growth and feed efficiency [9–11]. A meta-analysis of 13 broiler studies involving the use of this commercial blend showed that its inclusion in diets increased body weight gain and decreased feed conversion ratio and mortality [12].

The mechanism of action of phytochemicals is not clearly understood but may depend upon the composition of the active ingredients in the product being used. The beneficial effects of phytochemicals are attributed to their antimicrobial and antioxidant properties. In addition, the inclusion of phytochemicals in the diets alters and stabilizes the intestinal microbiota and reduces microbial toxic metabolites in the gut, owing to their direct antimicrobial properties on various pathogenic bacteria, which results in relief from intestinal challenge and immune stress, thus improving performance [13]. Another important beneficial effect of dietary inclusion of phytochemicals is reduction in oxidative stress and increase in antioxidant activity in various tissues, and thus, improved health [14]. Phytochemicals also exert their action through immunomodulatory effects such as increased proliferation of immune cells, modulation of cytokines, and increased antibody titers [5–8, 15–18]. In addition, phytochemicals in *Allium hookeri* improved gut barrier function, as demonstrated by increased expression of gut tight junction proteins in the mucosa of lipopolysaccharide (LPS)-treated young broiler chickens [18].

## Examples of phytochemical antibiotic alternatives in poultry and livestock production

### Dietary phytochemicals enhancing innate immunity in poultry

A growing body of scientific evidence has demonstrated that many of the health-promoting activities of phytochemicals are mediated through their ability to enhance host defense against microbial infections [4, 19]. The immune-activating properties of medicinal plants such as dandelion (*Taraxacum officinale*), mustard (*Brassica juncea*) and safflower (*Carthamus tinctorius*) have been evaluated in vitro using avian lymphocytes and macrophages [9]. All three extracts inhibit tumor cell growth, stimulate innate immunity and exert antioxidant effects in poultry [9]. Beneficial effects of cinnamaldehyde ((2*E*)-3-phenylprop-2-enal), a constituent of cinnamon (*Cinnamomum cassia*), a widely used flavoring compound that has been traditionally used to treat human diseases, has been investigated. Cinnamaldehyde stimulated primary chicken spleen lymphocyte proliferation in vitro and activated macrophages to produce high nitric oxide (NO) [6, 9].

Because of increased regulation of AGPs in poultry production, control of enteric diseases such as necrotic enteritis (NE) and coccidiosis, which have been traditionally controlled by in-feed antibiotics [2], needs antibiotic-free disease control strategies. Although plant-derived chemicals with potent medicinal properties are currently in clinical trials for treatment of a variety of diseases in humans, only limited research has documented the beneficial effects of phytochemicals on avian diseases [4, 19]. Dietary supplementation of 1-day-old chickens with cinnamaldehyde at 14.4 mg/kg showed up to 47-fold greater levels of gene transcripts encoding interleukin (IL)-1β, IL-6, IL-15 and interferon (IFN)-γ in intestinal lymphocytes, compared with chickens given a standard diet [15, 19]. Cinnamaldehyde-fed chickens showed 17 and 42% increased body weight gains following *Eimeria acervulina* and *E. maxima* infections, respectively, 40% reduced *E. acervulina* oocyst shedding, and 2.2-fold higher *E. tenella*-stimulated parasite antibody responses, compared with the control. The most reliable genetic network induced by dietary cinnamaldehyde treatment is related to antigen presentation, humoral immunity, and inflammatory disease. Chickens continuously fed 15 mg/kg anethole from hatch and orally challenged with live *E. acervulina* oocysts showed increased body weight gain, decreased fecal oocyst excretion, and greater anti-parasite serum antibody responses, compared with the control group. Global gene expression analysis by microarray hybridization in the intestinal lymphocytes of anethole-fed birds showed that many genes related to the inflammatory response are altered [17]. The levels of transcripts encoding IL-6, IL-8, IL-10 and TNF superfamily member 15 (TNFSF15) in intestinal lymphocytes were increased in parasite-infected chickens given the anethole-containing diet, compared with the control chickens given a standard diet.

Garlic metabolites also have been tested in poultry using propyl thiosulfinate (PTS) and propyl thiosulfinate oxide (PTSO) [16]. Supplementation of 10 mg/kg PTS/PTSO increased body weight gain and serum antibody titers against profilin, an immunogenic protein of *Eimeria*, and decreased fecal oocyst excretion in *E. acervulina*-challenged chickens compared with chickens fed a control diet [16]. The addition of PTS/PTSO in broiler’s diet altered many genes related to innate immunity, including TLR3, TLR5 and NF-κB [16] and down-regulated expression of IL-10 compared with the control diet. In uninfected chickens, dietary supplementation with PTS/PTSO increased the levels of transcripts encoding IFN-γ, IL-4, and an antioxidant enzyme, paraoxonase 2, but decreased transcripts for peroxiredoxin-6 [16].

Combination of multiple phytochemicals exert synergistic effects to reduce negative consequences of enteric infections. Dietary supplementation of newly hatched broiler chickens with a mixture of *Curcuma longa*, *Capsicum annuum* (pepper), and *Lentinus edodes* improved body weight gain and serum antibody titers against profilin, and reduced fecal oocyst shedding in *E. acervulina*-infected birds, compared with the birds fed the control diet or a diet containing *Capsicum* plus *Lentinus* [5]. The effects of carvacrol, cinnamaldehyde and *Capsicum* oleoresin on the regulation of expression of genes associated with immunology, physiology, and metabolism have been investigated in chickens using high-throughput microarray analysis [15]. The levels of transcripts for IL-1β, IL-6, IL-15 and IFN-γ in gut lymphocytes were also greater in the *Curcuma*/*Capsicum*/*Lentinus*-fed birds, compared with those fed the standard, *Curcuma* or *Capsicum*/*Lentinus* diet. In a follow-up study, a combination of carvacrol, cinnamaldehyde and *Capsicum* oleoresin, or a mixture of *Capsicum* and *Curcuma* oleoresins increased protective immunity against experimental *E. tenella* infection following immunization with profilin, compared with untreated and immunized controls [10]. Immunized chickens fed the carvacrol/cinnamaldehyde/*Capsicum*-supplemented diet showed increased numbers of macrophages in the intestine, while those given the *Capsicum/Curcuma* oleoresin-supplemented diet had increased numbers of intestinal T cells, compared with untreated controls. While numerous studies have shown disease prevention or immune-enhancing effects of phytochemicals, few have examined the underlying mechanisms that are involved. Some phytochemicals inhibit innate immune response by targeting pathogen pattern recognition receptors or their downstream signaling molecules [20].

The *Clostridium*-related poultry disease such as NE causes substantial economic losses on a global scale [21]. It has been suggested that dietary phytonutrients could be used against NE. Supplementation of a mixture of *Capsicum* and *Curcuma longa* oleoresins (XTRACT^®^) from hatch increased body weight and reduced gut lesion scores in NE-afflicted birds, compared with infected birds given the non-supplemented diet [7]. The XTRACT^®^-fed birds also had lower serum α-toxin levels and reduced mRNA expression of IL-8, lipopolysaccharide-induced TNF factor (LITAF), IL-17A and IL-17F in intestine, but increased cytokine/chemokine levels in splenocytes, compared with birds fed with the control diet. This study documented the molecular and cellular immunity changes following dietary supplementation with extracts of *Capsicum* and turmeric that may be relevant to protective immunity against avian NE [7]. Future studies are needed to define the molecular and cellular mode of action of this phytochemical combination for the control of NE in the field.

### Dietary phytochemicals on weaning pig health

Phytochemicals have been used for human nutrition and health improvement due to their potential biological functions, such as, antiviral, antimicrobial, antioxidant and anti-inflammatory effects [2, 5, 22]. Various phytochemicals exhibit a wide spectrum of antibacterial activities against Gram-negative and Gram-positive bacteria [23] with several different modes of action. First, phytochemicals directly kill bacteria due to their hydrophobicity, which enables them to partition into the lipids of the bacterial cell membrane and mitochondria, resulting in leakage of critical intracellular materials [24]. Second, phytochemicals contain a high percentage of phenolic compounds, which possess strong antibacterial properties [25]. Third, the active components in phytochemicals disturb the enzyme system of bacteria and block their virulence [26]. Fourth, certain bioactive components in phytochemicals may prevent the development of virulence structures in bacteria, such as flagella, which critical for bacterial adhesion [27].

Phytochemicals are also proposed for use as antioxidants in animal feed, which will protect animals from oxidative damage caused by free radicals. The antioxidative properties of extracts of oregano, thyme, clove, pepper, lavender and basil have been evaluated by many studies in vitro [28, 29]. Our recent in vitro assays have also revealed that EOs extracted from peppermint and spearmint have cellular antioxidant activities by increasing intracellular glutathione concentration in H_2_O_2_-stimulated intestinal epithelial cells (unpublished data). Frankič et al. [30] showed that supplementation of phytochemicals to pigs reduced DNA damage in lymphocytes, which indicates their potentially beneficial effects on the immune system under dietary-induced oxidative stress. The antioxidant activity of phytochemicals is highly correlated with their chemical composition [31]. Phenolic OH groups in thymol, carvacrol and other phytochemicals act as hydrogen donors to the peroxy radicals produced during the first step in lipid oxidation, thus retarding H_2_O_2_ formation [32].

The anti-inflammatory effects of phytochemicals have been widely reported in in vitro cell culture models. EOs from clove, tea, garlic, cinnamon and others have potential anti-inflammatory activities and suppress the production of TNF-α, IL-1β and NO from LPS-induced mouse macrophages [33]. Our previous research in vitro with porcine alveolar macrophages showed that carvacrol, *Capsicum* oleoresin, cinnamaldehyde, garlic, eugenol, anethol, and turmeric oleoresin suppress the production of proinflammatory cytokines (TNF-α and IL-1β) from LPS-stimulated macrophages [22], which indicates that all of these phytochemicals have anti-inflammatory effects. The modes of action for the anti-inflammatory activities of phytochemicals are not clear, but evidence suggests that these effects are partially mediated by blocking the nuclear factor (NF)-κB activation pathway [34]. For example, curcumin can block cytokine-induced NF-κB DNA binding activity, RelA nuclear translocation, IκBα degradation, IκB serine 32 phosphorylation, and IκB kinase activity [34].

Weaning is one of the most challenging and critical stages in swine production. Its effects are multifactorial, including behavior, environment, disease, immunity and nutrition. In this period, piglets are immediately subjected to a combination of stressors that predispose them to diarrhea, which can adversely affect survival at an early and most vulnerable stage [35]. The beneficial effects of phytochemicals on weaning pigs have been reported by different research groups. Manzanilla et al. [36] and Nofrarías et al. [37] have suggested that phytochemicals improve gut health. They have reported that a mixture of phytochemicals (XTRACT^®^) standardized to 5% (w/w) carvacrol, 3% cinnamaldehyde, and 2% *Capsicum* oleoresin (oregano, cinnamon and Mexican pepper) increases stomach contents, suggesting an increased gastric retention time. In addition, XTRACT^®^ decreases ileal total microbial mass and increases the lactobacilli:enterobacteria ratio. Michiels et al. [38] have also indicated that supplementing with 500 ppm carvacrol and thymol reduces the number of intraepithelial lymphocytes and increases villus height/crypt depth in the distal small intestine.

*Escherichia coli* post-weaning diarrhea is a common cause of death in weaned pigs. This diarrhea is responsible for economic losses due to mortality, morbidity, decreased growth performance, and cost of medication [39]. Enterotoxigenic *E. coli* are the most dominant types of pathogenic *E. coli* that cause diarrhea in both pre- and post-weaning piglets [40]. *Capsicum* oleoresin, garlicon, and turmeric oleoresin have been tested in an in vivo pathogenic *E. coli* challenge study to determine the effects of individual phytochemicals on diarrhea and gut health of weaning pigs [41]. The pigs were weaned at 21 days of age, transported to the experimental facility, and given the experimental diets immediately. After a 5-day adaptation period, they were challenged with three consecutive daily doses of 10^10^ colony forming units/3 mL of a hemolytic *E. coli* with F18 fimbria. The experimental diets were a control diet based on corn and soybean meal and three additional diets containing 10 mg/kg of each plant extract. The *E. coli* infection increased diarrhea score, frequency of diarrhea, and reduced growth rate, feed efficiency and villus height of the small intestine. However, supplementation with individual phytochemicals reduced overall frequency of diarrhea of pigs, indicating that feeding phytochemicals may enhance disease resistance in pigs. Supplementation with phytochemicals also improved ileal villus height and upregulated mRNA expression of the *MUC*-*2* gene, which indicated that the reduced diarrhea score was likely due to improved gut barrier function and integrity. Pigs infected with *E. coli* showed an increased number of white blood cells, serum proinflammatory cytokine (TNF-α) and acute phase protein (haptoglobin) and increased recruitment of macrophages and neutrophils in the ileum. Dietary supplementation with phytochemicals reduced white blood cells, neutrophils, serum TNF-α and haptoglobin and the numbers of macrophages and neutrophils in the ileum compared with the control diet. These observations indicate that feeding low doses of phytochemicals reduces both systemic and local inflammation caused by *E. coli* infection.

To decipher the underlying mechanism behind the benefits of feeding phytochemicals, microarray analysis has been conducted to characterize gene expression in the ileal mucosa of pigs experimentally infected with *E. coli*. Microarray results indicate that feeding phytochemicals enhances the integrity of membranes, especially several tight junction proteins. Supplementation of phytochemicals downregulates expression of genes related to antigen processing and presentation and other immune-response-related pathways, indicating that these phytochemicals attenuate the immune responses caused by *E. coli* infection [42].

Another in vivo study on porcine reproductive and respiratory syndrome virus (PRRSV) [43] showed that feeding *Capsicum* oleoresin, garlicon, and turmeric oleoresin to weaning pigs enhances the immune responses to PRRSV challenge and may help alleviate the negative impact of infection, as indicated by reduced viral load and serum concentrations of inflammatory mediators, and shortened duration of fever. In summary, phytochemicals are strong candidates to replace antibiotics to improve growth performance and health of pigs. The potential benefits of plant extracts may differ due to the large variation in the composition of plant extracts. This diversity prompts us to select optimal feed additives for evaluating their possible roles as alternatives to antibiotics in swine production.

### Use of phytonutrients in ruminants

In ruminants, the host and rumen microorganisms establish a symbiotic relationship by which the animal provides nutrients and the proper fermentation conditions, and microbes degrade fiber and synthesize microbial protein as an energy and protein supply for the host, respectively. Carbohydrates are fermented in the rumen into pyruvate, resulting in the production of metabolic hydrogen. Volatile fatty acids (VFAs) are natural hydrogen sinks that help maintain the equilibrium of hydrogen and the fermentation process active. Retention of energy from glucose is the highest in propionate (109%), intermediate in butyrate (78%) and the lowest in acetate (62.5%). Although methane is effective in retaining hydrogen, the energy retained is lost through eructation and not available to the host. Manipulation of the relative proportions of these VFAs is key to the development of targets to modify rumen microbial fermentation [44]. Protein degradation is also important for the supply of nitrogen to rumen microbes for their growth, but excess ammonia nitrogen is absorbed through the rumen wall, transformed into urea in the liver, and excreted through the urine. In most production systems, ammonia nitrogen in the rumen is produced in excess of the ability of rumen microbes to use it, resulting in significant production costs and an increase in the release of nitrogen into the environment [45]. Therefore, controlling proteolysis, petidelysis and deamination should also be considered targets of interest in the modulation of rumen fermentation [44]. In fact, in a recent study, Van der Aar et al. [46] indicated that improving the efficiency of the digestion processes in ruminants is still the most efficient strategy to improve animal performance.

AGPs are efficient in shifting rumen fermentation towards more efficient energy and nitrogen utilization pathways [47], improving productivity in dairy and beef diets [48, 49]. Therefore, industry is searching for alternative feeding strategies and/or additives that will allow it to maintain the current level of production without increasing the cost.

Phytonutrients are a group of small organic molecules present in plants that modify the nutritional value of feeds by either modulating the digestion of nutrients in the digestive tract, or other systemic metabolic pathways. Some phytonutrients have a strong antimicrobial activity [50]. However, these molecules are not suitable for use in ruminants because the activity of rumen bacteria is essential for the proper function of the rumen. Research on alternatives to antibiotics as feed supplements in cattle should focus on molecules and doses that are able to produce subtle changes in the microbial metabolism and modify their rate of growth [51]. In the context of the continuous flow in the rumen, a change in growth rates results in changes in the proportion of rumen bacteria populations, resulting in changes in the fermentation profile. For example, Patra and Yu [52] were able to prove how different phytonutrients have different capacities in modifying the structure of the microbial population of the rumen. These changes are large in oregano (where thymol and carvacrol are the main active components) and peppermint (where menthol and menthone are the main active components) oils, but smaller, and more adequate, in clove bud (where eugenol is the major active component) and garlic oils. Ferme et al. [53] also have demonstrated that the reduction in protein degradation and ammonia production is achieved through changes in the total amount of *Prevotella* ssp. in the rumen; a major group of bacteria involved in amino acid deamination. These findings are important to set clear objectives in the search for alternatives to AGPs, which should identify phytonutrients that can modify the VFA proportions and protein degradation in the rumen without affecting nutrient degradation and the normal function of the rumen.

Most phytonutrients of interest in animal feeding are classified into three main groups: saponins, tannins and EOs. Saponins and sarsaponins are the main active components of several phytochemicals, including yucca, quillaja, alfalfa and fenugreek. Saponins exhibit antibacterial [54] and antiprotozoal [54, 55] activity, resulting in a reduction in ammonia nitrogen concentration. Tannins are phenolic compounds found in almost every plant part, and are divided into two groups, hydrolysable and condensed tannins. Condensed tannins have the ability to bind and precipitate proteins and may be useful in the control of protein utilization by ruminants [56], but at high levels may interfere with dry matter (DM) intake and digestibility of nutrients [56], and may decrease the incidence of bloating [55]. EOs are secondary plant metabolites present in many plants and may have a wide range of effects. In this section, we review recent research on the use of EOs as feed additives in ruminants.

#### Essential oils as modifiers of rumen fermentation

The increased rumen fermentation is indicated by the increase in propionate and decrease in methane, acetate and ammonia nitrogen, without reducing total VFA [57] in the in vitro fermentation system. When phytochemicals are tested, a considerable variation in fermentation with different extracts is observed due to the content of active compounds in these extracts [58]. Therefore, it is necessary to either report the concentration of these active compounds in phytochemicals, or use the active components to define activities, doses and mechanisms of action in an unequivocal form.

For example, garlic oil reduces the proportions of acetate and branched-chain VFAs, and increases the proportions of propionate and butyrate in vitro [57, 59], and the fermentation profile is consistent with changes observed when methane inhibitors are supplied to ruminants. The anti-methanogenic effect of garlic and its active components is the result of direct inhibition of Archea microorganisms in the rumen through the inhibition of hydroxymethylglutaryl coenzyme A (HMG-CoA) reductase; a specific pathway essential for the membrane stability of Archea [57, 59]. This observation was supported by Miller and Wolin [60], who reported similar effects when using statins, known to inhibit HMG-CoA reductase. However, benefits are often inconsistent, and strong inhibition of VFA production by garlic oil has been reported in some cases [59, 61, 62]. The variable effects of garlic oil on total VFA production is likely due to the short margin of safety in the doses between adequate and toxic levels.

Cinnamaldehyde and eugenol also reduce the molar proportion of acetate, and increase the molar proportions of propionate and butyrate [59, 61]. These observations are consistent with improved energy retention by those phytochemicals and potentially due to the inhibition of methanogenesis [63]. Cinnamaldehyde also reduces ammonia nitrogen and increases free amino acids, suggesting that deamination of amino acids is inhibited in the rumen [59, 61]. Ferme et al. [53] have reported that cinnamaldehyde reduces *Prevotella* spp., bacteria involved in deamination, in an in vitro rumen simulation system. However, Eugenol inhibits the breakdown of large peptides to amino acids and small peptides [59]. The combination of eugenol and cinnamaldehyde may work in synergy to inhibit peptidolysis and deamination, and then improve the overall supply of amino acids and small peptides to microorganisms and the host. Therefore, a synergetic advantage could be expected by combining specific phytonutrients that work at different levels in the same metabolic pathway.

There are limited data reported about the effects of phytochemicals on performance of ruminants. Feeding cinnamaldehyde alone or in combination with eugenol results in increased in milk production of 1.7–2.7% [64]. An even better response is reported when a combination of cinnamaldehyde, eugenol and capsicum is fed to dairy cattle, with increases in energy-corrected milk production of 5.2% [65] and 3.2% [66]. However, no differences have been observed in most of cases due to the small size of the studies. Bravo et al. [67] have summarized a large set of in vivo field trials using combinations of cinnamadehyde and eugenol through a meta-analysis, and have reported an improvement in milk production of 3.0% for dairy cattle.

#### Essential oils as modifiers of metabolic activities

Many phytonutrients have metabolic effects that are not related to their activities in the rumen [68, 69]. Preliminary in vitro rumen fermentation studies in dairy cattle have not identified capsicum as a potential modifier for rumen function [61, 70]. Capsicum increases DM and water intake in beef cattle from 9.2 to 14% [70–72], while these effects are not observed in dairy cattle [73, 74]. The benefits may be more significant when intake is compromised, such as when the cattle arrive at feedlots or during heat stress. The increase in DM intake patterns is probably also related to a more stable rumen pH [75].

Capsicum has been reported to modulate immune function [42]. Oh et al. [76] have reported an improvement in immunity indicators, with an increase in neutrophils and decrease in lymphocytes when cattle are fed rumen-protected capsicum. Feeding rumen-protected capsicum is reported to improve milk production. Stelwagen et al. [77] and Wall et al. [78] have reported increases in milk production of 6.6 and 9.1% in pasture and intensive production systems, respectively. Another three studies have also reported that supplementation of rumen-protected capsicum improved milk production by 6.2% [76], 10% [79], and 4.4% [80], respectively. The average increase in milk production in those studies was higher than the effects attributed to the modulation of rumen fermentation. Oh et al. [80] observed that supplementation with rumen-protected capsicum resulted in a lower insulin concentration after a glucose tolerance test. These results suggest that capsicum modifies glucose metabolism, redirecting glucose away from peripheral tissues and towards the mammary gland to increase milk production. In fact, Bovine somatotropin (bST) increases milk production by an average of 13%, redirecting glucose to the mammary gland, although the mechanism of action is different. This is an exciting new application of phytonutrients that presents an opportunity to improve production, not only by reducing the use of antibiotics, but also by providing an alternative to the use of some hormones. The average effect of rumen modifiers like monensin, yeast or some phytonutrients, commonly increase milk production by 2–4%, while capsicum increases milk production by an average of 7%.

## Phytochemicals and the digestive microbiota

The mammalian gastrointestinal tract harbors a dense and diverse microbial community, which is composed primarily of bacteria but also includes fungi, Archaea and viruses. Collectively, these are referred to as intestinal microbiota. These microorganisms are environmentally acquired, and their metabolic functions can shape host physiology. Many vertebrates consume a diet rich in complex nutrients that are indigestible by their own intestinal enzymes, relying on the diverse biochemical catabolic activities of the microbiota. Available evidence strongly suggests that the gut microbiota plays important roles in host energy harvest, storage and expenditure, as well as overall nutritional status [81–84]. It must be highlighted that germ-free animals that lack any microbiota weigh less and have less fat than conventional animals [85], pointing out a key role of the microbiota in weight gain. Gut microbiota may affect weight gain through regulating nutrient extraction, and modulating the immune system and metabolic signaling pathways [82].

Many classes of substances with antibiotic activity that are effective for animal growth promotion display multiple modes of action and spectra of activity over the gastrointestinal microbiota. It has been difficult to predict which microbial changes are responsible for increases in weight gain, feed efficiency or health promotion. Culture-independent approaches using next-generation DNA sequencing have provided researchers with a revolutionary tool to look into microbiomes that could not be achieved before, and has begun to transform our view of intestine-associated biodiversity of animal production. Improving the understanding of microbiota and host metabolism would help to develop better strategies and products for animal production and welfare, food safety and public health. The selection of microbes that aid in nutrient extraction, regulating microbial carbohydrate, protein and lipid metabolism, and the prevention of subclinical infections will help to promote productive parameters [83].

The intestinal microbiota plays a critical role in inflammatory gut diseases of humans and animals [86]. Recent development and application of next-generation sequencing technologies using 16S rRNA gene have allowed investigation of the significant roles of the microbiota in gastrointestinal tract diseases, and have facilitated investigation of host–pathogen interaction in NE [86]. The effect of dietary phytochemicals on gut microbiota was studied in three major commercial broiler chickens fed with *Capsicum* and *C. longa* oleoresins [13]. Among the three chicken breeds, Cobb, Hubbard and Ross, oleoresin supplementation was associated with altered intestinal microbiota. The results suggested that dietary feeding of *Capsicum* and *C. longa* oleoresins reduces the negative consequences of NE, in part, through alteration of the gut microbiome. Although these are preliminary characterizations of the effects of dietary phytochemicals on gut microbiota but document the role of dietary *Capsicum* and *C. longa* oleoresins in regulating disease susceptibility to NE via altering the intestinal microbiota in commercial broiler chickens.

A recent study [13] showed that *Firmicutes* was the dominant phylum and *Lactobacillus* was the predominant genus identified in the ileum in all broiler breeds and all treatment groups. These results are consistent with previous studies that showed *Lactobacillus* as the principal microorganism in the gastrointestinal tract of uninfected conventional broilers [87]. Because *Firmicutes* are fat-loving Gram-positive bacteria [88] this result suggests an inter-relationship of these bacteria and genetic selection for fast-growing characteristics of these broilers by the industry. In a recent comparative study [13], changes in the proportion of intestinal lactobacilli, as well as the total number of operational taxonomic units (OTU) between the three commercial broiler breeds were observed. *Candidatus Arthromitus* is a group of non-cultivable, spore-forming, *Clostridium*-related, commensal segmented filamentous bacteria (SFBs) that colonizes in the digestive tracts of animal species, and has been identified in three commercial broiler breeds [89]. As the core OTU, *C. Arthromitus* has been identified in all three groups of the Cobb and Hubbard broilers [13]. The most intriguing feature of SFBs is their close interaction with epithelial cells in the terminal ileum and their intimate cross talk with the host immune system. *C. Arthromitus* belongs to gut-indigenous *Clostridium* that induce immune regulatory T (Treg) cells. Intestinal Treg cells express T cell receptors that recognize antigen derived from gut microbiota [90]. SFBs send signals to control the balance between IL-17-producing T helper (Th17) cells that sustain mucosal immunity, and forkhead box p3 in the intestine [90]. Our previous studies have also reported that chicken IL-17A transcripts increase in the duodenum and jejunum of *E. maxima*-infected chickens [13, 91] where early inflammatory response plays an important role for development of protection against *Eimeria* infection. Upon feeding a mixture of oleoresins from *Capsicum/C. longa*, there is a different shift in the bacterial community in all broiler breeds with NE. Therefore, co-infection with *E. maxima* and *C. perfringens* may influence the presence of *C. Arthromitus* and the host immune system in Ross chickens. It will be important to conduct further studies to investigate the functional immune modulatory effects of dietary phytonutrients on *C. Arthromitus* in genetically different broiler breeds. In conclusion, dietary phytonutrients exert beneficial effects on gut health to reduce the negative consequences of NE, and nutratherapeutics mechanism may involve altering gut microbial communities. Further studies on the effects of dietary phytonutrients on gut microbiota in commercial broiler breeds are needed to develop alternative ways to reduce or replace antibiotics in poultry disease control. Future studies on the role of the avian intestinal microbiome in immune regulation and host–pathogen interactions are expected to shed new light on the host response to NE that will be beneficial for practical poultry husbandry.

In foregut fermenters, such as cattle and sheep, up to 50% of their energy may be obtained from microbial metabolites [92], including VFAs. In contrast, hindgut fermenters (such as pigs and chickens), in which most fermentation takes place in the cecum and large intestine, receive only 5–10% of energy demands from microbial fermentation products [93]. Although these differences seem to be important from a functional point of view, in ruminants or monogastrics, gastrointestinal microbiota composition is similarly central to improved animal production in both groups, and the impact of phytochemicals on these microbiota might be responsible for most of the positive effects observed.

Many beneficial properties of plants are derived from their specific bioactive components, which are also synthesized as chemical protectants against microbial infection. The most important useful phytochemicals with antimicrobial activities can be divided into several categories, such as phenolics/polyphenols, terpenoids/essential oils, alkaloids, and lectins/polypeptides [94]. Some compounds among these categories are known to be important for improving animal production, as well as inducing an extensive number of health-promoting effects. Tannins and EOs are fed commercially to several domestic animal species and, as growth promoters, they modify the gut microbiota in different ways.

Tannins are a complex group of polyphenolic compounds found in many plants species, functionally defined by their capacity to complex macromolecules (proteins and polysaccharides) and metal ions, which are commonly included in ruminant diets such as forage and sorghum. Tannins are chemically classified as hydrolysable or condensed based on their chemical structure, and are widely used to improve several aspects of animal husbandry. Some tannins are potent antimicrobials, acting, for example, by iron deprivation or interactions with vital proteins such as enzymes [95] or bacterial cell wall proteins [96], displaying either bactericidal or bacteriostatic activities [97]. Gram-positive bacteria are particularly sensitive to tannins [98].

In ruminants, tannins modify the digestive processes not only by binding dietary protein (rumen bypass), but also through modulation of rumen microbiota and improvement of the growth of certain bacterial populations [99]. The effects of tannins on rumen microbiota may vary depending on the molecular nature of these polyphenols [99, 100]. The understanding of in vivo interactions between rumen bacteria and sources of plant tannins are limited.

Approximately 90% of total microbiota in the bovine rumen is composed by *Firmicutes* and *Bacteroidetes*, with large inter-individual variance in their relative abundance, with a strong inverse correlation between abundance of both phyla [101]. In steers fed with a high-starch diet, bacterial populations belonging to the *Bacteroidetes* were the most abundant in all animals (almost 50%) while *Firmicutes* accounted for ~40% of the total microbiota. However, this predominance was inverted when a blend of tannins were added to the feed, with a significantly higher percentage of *Firmicutes* and a reduction in *Bacteroidetes*. Accordingly, steers supplemented with tannins have a higher *Firmicutes* to *Bacteroidetes* (F/B) ratio in comparison with the control group [101]. Many studies have reported that F/B ratio increases when body mass index is increased, and F/B ratio is higher in obese than in lean animals [102–104]. The rational basis for the apparent relation between F/B ratio and increase in body weight is that *Firmicutes* are not as effective as *Bacteroidetes* at gathering energy from digesta for themselves, leaving more energy to be absorbed by the host.

Diversity of rumen microbiota is one of the key features in ruminant animals, which confers upon cattle the ability to adapt to a wide range of dietary conditions [105]. Dietary quebracho and chestnut tannins diminish rumen richness but do not significantly affect the complexity of the bacterial communities (i.e. balance between the relative abundances of bacterial taxa). There is an increase in rumen microbiota richness but no change in Shannon’s diversity index after supplementation with a blend of polyphenols and EOs in dairy heifers fed a high-grain diet, supporting the idea that polyphenols can modulate bacterial richness without disrupting the overall structure of the rumen microbiota population. Similarly, β-diversity analysis of rumen samples of steers fed with chestnut and quebracho showed no significant changes in bacterial diversity compared with the control group [101]. Low microbial richness in the rumen is closely linked to a higher feed efficiency in dairy cows [106]. The authors have suggested that lower richness in the rumen of efficient animals results in a simpler metabolic network, which leads to higher concentrations of specific metabolic components that are used to support the host’s energy requirements. Diversity analysis indicate that bacterial richness is lowered by tannins, but the overall bacterial complexity of the rumen is not significantly affected by chestnut and quebracho tannins supplementation.

Several studies have found an increase of rumen pH, decrease of ammonia concentration, and lower methane emissions after feed supplementation with several tannins including chestnut and quebracho, resulting in a reduction of protein degradation and therefore an improvement in nitrogen utilization in the rumen [107]. Tannins are considered as alternative agents to antibiotics, they improve animal health and productive performance while suppressing methanogenesis. These observations could be explained by changes in the microbiota in the rumen. Significant changes in the abundance of certain taxa have been detected in tannin-treated steers. Among Bacteroidetes, *Prevotella* was the most abundant genus, accounting for >40% of this phylum. The abundance of *Prevotella* was lower in tannin-supplemented animals than in the control group. In contrast, Clostridia was the predominant class, which accounted for >90% of total *Firmicutes*, and it was significantly enhanced in tannin-treated animals. Among Clostridia, Ruminococcaceae was the most abundant family and showed a significantly higher abundance in tannin-supplemented animals. Within the Ruminococcaceae, most of the sequences obtained in untreated animals belonged to unclassified members and the genus *Ruminococcus*, and both taxa were enhanced in tannin-treated steers. Other non-clostridial bacteria within the phylum *Firmicutes* were significantly altered by tannins, including members of class Erysipelotrichi. Members of class Bacilli (*Streptococcus* and *Lactobacillus*) showed moderate increases in their abundance in tannin-treated animals. Genus Fibrobacter was significantly affected by tannins, accounting for 0.10% of total microbiota in the control animals and only 0.005% in tannin-treated animals. Other minor fibrolytic bacteria were more abundant in tannin-treated steers, including the genus *Blautia* and member of the Eubacteriaceae genus *Anaerofustis*. Tannins remodel the bacterial ecosystem of the rumen, particularly the niche of fiber and starch degradation, and the methanogenic bacteria [108].

*Treponema* is also reduced by tannins. Among Veillonellaceae members, *Succiniclasticum*, which specializes in fermenting succinate to propionate, doubles its levels in tannin-treated animals. Lipolytic genus *Anaerovibrio* is significantly enhanced by tannins. *Selenomonas* is also increased in tannin-supplemented animals. Among ureolytic bacteria, *Butyrivibrio* is the most abundant and it is negatively affected by tannin treatment, as well as *Treponema* and *Succinivibrio*. Methanogens belonging to the phylum Euryarchaeota are less abundant in tannin-supplemented steers and their levels are inversely correlated with rumen pH. *Methanosphaera* is also reduced by tannins. Current literature indicates that tannins can be supplemented to improve the sustainability of both dairy and beef cattle by reducing methane emissions and nitrogen excretion, and enhancing animal performance.

In monogastrics, that is, broiler chickens, tannins obtained from several sources seem to improve growth performance and reduce the detrimental effects of pathogenic bacterial species such as *C. perfringens* [101]. The establishment of a stable microbiota is a complex process that is influenced by various factors, including genetic lineage, age, diet, use of growth promoter antibiotics, probiotics, litter composition, stress and disease [86, 109–111]. Therefore, any alteration in the intestinal microbiota may have functional consequences to the health of the host and, therefore, productivity.

The broiler chicken gastrointestinal tract is colonized by a dense community of microorganisms that is intimately connected to the global heath and development of the host. The cecum houses the highest microbial cell densities of the chicken gut and performs key process for birds such as the fermentation of cellulose, starch and other resistant polysaccharides [86]. A principal coordinate analysis (PCoA) based on unweighted UniFrac distances was conducted to determine any differentiation between sample clusters of tannin-treated versus antibiotic-growth-promoter-treated versus untreated birds. PCoA plots revealed that the samples corresponding to each dietary treatment shaped distinct series, suggesting that tannins differentially modulate cecal microbiota.

High-throughput sequencing of 16S rRNA gene amplicons has been used to identify functional diversity [112] or variability [113] of the microbiome in the gut of broiler chickens. In most studies related to tannins, cecal microbiota in chickens was dominated by *Firmicutes* and *Bacteroidetes* [114, 115], comprising >80% of the microbiota. The most abundant *Bacteroidetes* detected in cecal contents belonged to genus *Bacteroides* and an unclassified genus of the family Barnesiellaceae. Among the *Firmicutes*, order Clostridiales and family Ruminococcaceae were the most abundant taxa. The F/B ratio was significantly higher in tannin-fed animals than in the control or antibiotic growth promoter groups.

*Bacteroides* is a Gram-negative genus that utilizes plant glycans as its main energy sources. *Bacteroides* is one of the main bacteria involved in producing short-chain fatty acids (SCFAs) [116], and plays an important role in breaking down complex molecules to simpler compounds that are essential for host growth [117]. SCFAs are absorbed by the host and used as an energy source but also have a variety of distinct physiological effects. SCFAs are saturated aliphatic organic acids that consist of 1–6 carbons of which acetate, propionate and butyrate are the most abundant (≥95%). Although *Bacteroides* generates acetate and propionate, its ability to produce butyrate has not been reported. Order Clostridiales are generally known as important contributors to short-chain fatty acid (SCFA) metabolism [86] because it contains a variety of bacterial families, among which Ruminococcaceae and Lachnospiraceae are capable of fermenting various substrates to butyrate. Feed tannin supplementation of chickens decreases the abundance of *Bacteroides*, which could reduce acetate and propionate production. However, it would be compensated by an increase in Clostridiales, particularly Ruminococcaceae, with a possible increase in butyrate production [96]. Concordantly, Masek et al. [118] have reported a global increase in SCFA production in poultry treated with tannic acid.

Lactic acid bacteria, which are usually associated with enhanced gut health and productivity, are interesting. It was reported that cecal microbiota contained lower proportions of *Lactobacillus* in AGP-fed chickens, compared with chickens in tannin and control groups [119–121]. Lactic acid bacteria, especially *Lactobacillus* strains, have been considered as probiotic microorganisms because of their activities in reducing enteric diseases and maintaining poultry health [122–124]. The presence of *Lactococcus* spp. has been correlated with weight gain [125].

The inclusion of different AGPs in diet influences the diversity of gastrointestinal microbiota. These changes would probably be one of the most important driving forces resulting in efficiency improvement of animal production. Similarly, the existing information clearly shows a significant alteration in the relative abundance of specific bacterial populations by some phytochemicals in the gut of domestic animals (13). These phytochemicals added to feed are also connected with higher productivity parameters. Therefore, these natural compounds are able not only to improve animal health and welfare directly, but also to modulate gastrointestinal microbiota and increase the impact on health and production. We are just barely starting to understand the dynamics between the highly complex connection between environment, host and microbiota. More information is necessary to clarify how we can manipulate gastrointestinal microbiota to increase animal productivity under diverse productive settings.

## Examples of commercial phytochemicals and their synergistic action with other feed additives

### Tannins in animal husbandry

Tannins are present in many feeds such as fodder legumes, browse leaves and fruits. Although the structure of tannins are chemically diverse, they have one unifying property: tannins bind proteins. During the last 30 years, tannins have been successfully used in animal production to improve health and productivity, and several products based on blends of particular amounts of hydrolysable (predominantly chestnut) and condensed (mostly quebracho) tannins were developed to take advantage of the benefits of each tannin in livestock. These products are being used in many countries to improve quality and production of milk, meat and eggs. In poultry, a blend of tannins can be added to feed at a final concentration of 0.5–1 kg/tonne, both in pre-mix or directly into feed, to obtain several benefits including reduction of mortality rate, improvement of feed efficiency, weight gain and intestinal health, reduction of NE and foot-pad lesions, and increased feces consistency and litter quality of commercial settings. The selected blend of tannins added to the diet stabilizes and increases feed intake according to reduction of taste variation by changes in feed formulation [126], and reduces feed stress by improving the flavoring characteristics. The distinctive antispasmodic effects of tannins that modulate gut motility [127, 128], with strong antibacterial effects on several pathogenic bacterial species and viruses [97, 129], as well as their toxins [97], are used to prevent and control enteric diseases, including several diarrheal diseases [130] and NE [96]. Reduction of enteric diseases, intestinal motility and bacterial load, concurrently with an increase of feed digestibility, produces a reduction of humidity in the litter, affecting directly animal health and welfare. It has become obvious when foot-pad disorders are observed in commercial farms, dietary tannins reduced up to 50% of the animals with lesions, and up to 20% reduction of animals with the most severe lesions.

These blend of tannins are also being used efficaciously to reduce the incidence of sub-clinical NE, and a slightly different blend is able to strongly reduce intestinal lesions in chickens on farms with a history of severe NE outbreaks. In experimental conditions, the tannin blend is able to reduce the most severe lesions as well as the number of animals with lesions. This result is also observed in commercial farms of different European, American and Asian countries where NE is a problem to different degrees. As an example, an integrated company in Brazil with a persistent history of sub-clinical NE started using the tannin product in 2015 and reduced the number of animals with lesions by 10%, improving productivity by almost 3% (Dr Joao Battista Lancini, personal communication).

A comparative analysis of AGPs versus tannin blend use in feed was carried out in a commercial trial in Argentina over a period of 13 months (5 cycles) in a poultry farm of ~200 000 animals. The farm was divided into six barns under regular commercial feed; three were fed with AGPs in feed and three with 0.1% blend of tannins in feed but without AGPs. Greater improvements in intestinal health, microbiological quality and humidity of litters, mortality rate, undigested feed, foot-pad lesions, and weight gain were observed in the animals treated with tannins versus antibiotics. Analysis of the results showed a positive difference of almost 10 points for the Production Efficiency Factor for the blend of tannins against AGPs in feed, showing the benefits of using these blend of tannins during different weather conditions throughout the year [131]. Tannins added in feed to improve productivity in combination with other products, including EOs, organic acids, probiotics and AGPs, have been used frequently by different companies in several countries with significant positive results (Dr Javier Quintar and Dr Joao Battista Lancini, personal communication).

In cattle, historically low doses of quebracho and chestnut tannins have been used in feed by many producers around the world to improve bypass protein from rumen degradation. Rumen bypass protein is one of the strategies to increase the amount of protein that enters abomasum and hence increases ruminant productivity. The reduction in protein degradation in the rumen may occur by the formation of a reversible tannin–protein complex in the rumen pH and/or the modulation of rumen microbiota. The addition of such tannins to a diet reduces the fermentability of protein nitrogen in the rumen [132]. Consequently, the flow of dietary amino acids into the duodenum of ruminants could be increased, as well as the total duodenal amino acid flow if ammonia nitrogen requirements for microbes could be met by supplementation of urea or ammonia salts.

In addition, added tannins are also used to prevent acidosis and bloating [133], modulate rumen microbiome to improve feed utilization [130], and reduce methane emissions [134] and nitrogen excretion [135]. A particular tannin mix added in feed was able to reduce liver abscesses in beef cattle by >80% [136]. Supplementation of tannin also reduced fecal moisture, resulting in better fecal consistency.

According to Rivera-Mendez et al. [137], the addition of up to 0.2% of a blend of tannin to steers during the feedlot finishing phase increased average daily gain by 6.5%. Body weight in young animals was improved up to 7% in commercial conditions before the breeding period [107, 138]. Similarly, DM intake tended to increase with level of tannin. Tannin supplementation increased gain efficiency (5.5%) and dietary net energy (3.2%). These results have been also observed in commercial feedlot finishing settings. The analysis of 15 different trials in North America between 2010 and 2013 using tannins at 0.25%, with or without antibiotics or ionophores in feed, showed an average daily gain of 9.2% and gain efficiency of 5.07% compared to non-tannin controls [139, 140]. Similar results have been observed in feedlots in other parts of the world, including large beef producers in Brazil [141, 142] and Argentina [136].

In conclusion, the addition of low-dose tannins to ruminant diets in intensive fattening is an available tool to increase nutrient use efficiency, improving daily weight gain and feed conversion, through different metabolic mechanisms. The estimated level of animal feed supplemented with tannins produced in the world in 2016 was 15 000 000 tonnes, reflecting the acceptance of tannins as an important tool in animal husbandry. The available scientific information about mechanism of action, the observed animal response and the accumulated experience in the use of tannins as feed additive confirms that tannins are a valuable alternative to complement or replace the use of AGPs in industrial livestock production.

### Synergistic action of phytochemicals with other feed additive antibiotic alternatives for commercial products

Designing an antibiotic alternative to address several components of gut health may work better than using a single approach to reduce negative consequences of gut damage caused by complex etiologies such as those that cause diseases such as NE. *C. perfringens* produces several exotoxins, including α-toxin and NE toxin B (NetB), that disrupt the intestinal epithelium, causing necrotizing lesions that constitute the characteristic sign of NE [21, 143].

For complex disease like NE, it takes a multi-faceted approach to decrease the effects of disease on gut health. For example, a commercial product Varium^®^ was designed to improve barrier function by removing pathogens by agglutination, removing biotoxins via adsorption, priming immune development, and providing energy to the enterocytes [144]. Varium^®^ has been tested in vitro for its ability to bind biotoxins of pathogenic bacteria (i.e. *C. perfringens* and *E. coli*) such as α-toxin, NetB toxin, lipopolysaccharide, heat-labile toxin and Shiga-like type 2 toxin. The binding of these toxins was dose dependent, with the exception of NetB toxin, which was bound 100% across the doses tested.

Two large broiler trials have been conducted to test the hypothesis that CaMM, or its blends with other materials (e.g. fermentable fibers, organic acids, and/or phytonutrients) could improve gut health and decrease the negative effects of avian NE. The two trials evaluated CaMM-based dietary products on growth performance, clinical signs, immunopathology, and cytokine responses of young broilers using disease challenge models with avian NE [144]. When tested in unchallenged birds, Varium exerted an effect similar to an in-feed AGP on body weight, feed intake, and FCR. Chickens fed a diet supplemented with CaMM plus a fermentable fiber and an organic acid showed increased body weight gain, reduced gut lesions, and increased serum antibody levels to *C. perfringens* α-toxin and NetB toxin compared with chickens fed the basal diet alone. Levels of transcripts for inflammatory cytokines such as IL-1β, IL-6, inducible NO synthase, and TNFSF15 were significantly altered in the intestine and spleen of CaMM-supplemented chickens compared with unsupplemented controls [144]. In Trial 2, Cobb/Cobb chickens were fed an unsupplemented diet or a diet supplemented with CaMM; each with a fermentable fiber and an organic acid, and co-infected with *E. maxima* and *C. perfringens* under subclinical infection conditions to elicit NE. Compared with unsupplemented controls, broilers fed with CaMM plus a fermentable fiber and an organic acid showed increased body weight gain, reduced FCR, mortality, and intestinal lesions, compared with chickens fed an unsupplemented diet.

Based on both broiler trials, it is recommended that dietary supplementation of CaMM or CaMM plus a fermentable fiber and an organic acid is useful to decrease negative effects of avian NE in the field. Future studies are needed to characterize further the CaMM-regulated physiological and immunological mechanisms that are activated in response to avian NE.

### Antibiotic alternatives: industry perspective

In general, there is a lack of consensus on what is meant by the phrase “antibiotic alternatives”. AGP use is a common practice that has been around for >65 years in modern livestock production that to this day has no consensus about its mechanism of action. Yet, most of the technologies discussed here have proposed or known mechanisms of action that involve inhibition, alteration or killing of one or more bacteria. In general, it appears that most people equate the phrase with something not termed an antibiotic that can be substituted for low level feeding of broad-spectrum antibiotics used to promote growth in livestock. The reason there is a need for alternatives to AGP is the recognition that the practice can lead to development of infective bacteria that are resistant to many of the current antibiotics available to human medicine. The rising incidence of superbugs globally and the rising human deaths from multiple drug-resistant bacteria have alerted WHO, CDC and UN to release strict action plans on reducing the use of antibiotics in animal production.

Regardless of which side of the argument over whether AGP use in animals is contributing to the problem of resistant bacteria in humans you are on, the sociopolitical momentum has created a marketing opportunity for selling meat from animals claimed to have never received antibiotics during production. This in turn creates a market for products that can provide the benefit of AGPs but not be antibiotics used in human medicine, or sometimes any antibiotic at all. The alternative to antibiotics market is growing rapidly and attracting interest from companies and organizations of all sizes and capabilities. This is evident from the need for a meeting such as this and the plethora of products marketed, with or without credible data, to be alternatives to AGPs. Although the banning of AGPs has accelerated over the last few years, the search for alternatives started in earnest following the ban in the EU of avoparcin in 1997.

The most important development in the search for credible alternatives is the increasing understanding in both human and veterinary medicine that the gastrointestinal tract is more than a nutrient-absorbing organ, but in fact is fundamental to health and development of humans and animals. The scientific advancement in our understanding of the importance of the gut environment and its barrier function in health provide a way to develop products that can deliver the benefits of AGPs without causing an increase in the emergence of antibiotic-resistant bacteria. This can be accomplished by using multiple technologies to maintain or strengthen gut barrier function. Scientific principles should be applied to the development of products such that they provide reliable positive benefits to the target animals.

In a recent survey, more than 70% of animal feed companies showed interest in willingness to use somekind of feed additive as antibiotic alternatives. However, there are still many challenges remaining with the most consistent concerns being consistency, safety and solid scientific proof. This is not surprising when you consider most of the popular alternative products marketed today modify the microbiota in some way to enrich beneficial bacteria. We are just learning what the desirable microbiota is and how it works in given animal, and we have even less knowledge of the variations between different animals and the normal daily and lifetime changes in different ecosystem. So, it is likely that a product that can deliver consistent results will need to incorporate two or more components that have complimentary and/or synergistic mechanisms of action. In addition to the microbiota, it will be necessary to understand clearly what impact the product has on the gut barrier which comprises the mucus layer, endothelial cells and attendant immunological cells and structures associated with the gut wall.

This is a relatively new field of research and as time goes on, the industry, through application of good science, will learn more. This will be both in the basic understanding of the gut environment, including the microbiota and the dynamic function of the gut barrier, and how to manipulate these structures in individuals, but as part of a population. Because it is new and there are many unknowns, regulation of these products poses a challenge in different regions of the world. What constitutes acceptable efficacy and what types of claims can be supported are largely unknown. However, there is little doubt that use of the FDA drug approval process is not a viable option today. Perhaps as science defines ways to measure and test efficacy in a consistent manner across several mechanisms of action, a regulatory pathway can be established. There will need to be tolerance and flexibility in the approval process for these products or the market will be flooded by products with no proof of efficacy or safety. At a minimum, these products should have scientific proof of efficacy in the target species for which they are marketed. In vitro tests are insufficient to provide confidence that a product will work in an animal, let alone provide consistent value across a population of animals.

## Conclusions and future directions

Increasing concerns about the increase of superbugs and limited development of new drugs for livestock and humans necessitates the timely development of alternatives to AGPs. With increasing availability of many different categories of antibiotic alternatives in the market for animal agriculture with various claims and efficacy, the industry needs to understand the mode of action associated with different types of antibiotic alternatives and the kind of synergy that can be offered by the combinations of different antibiotic alternatives, especially for prevention and treatment of complex diseases such as necrotic enteritis. Furthermore, the definition of the phrase antibiotic alternatives should be better defined, although this terminology is now an accepted term to refer to non-antibiotic substances that can be substituted for low-level feeding of broad-spectrum antibiotics that promote growth in livestock. Antibiotic alternatives will be mainly used to replace AGPs whose primary function is to decrease microbial populations and promote growth via many different modes of action that may include alteration and/or inhibition of microbial growth, decrease of inflammation, enhancement of innate immunity, reduction of oxidative stress, and improvement of gut integrity. Increasing marketing opportunity for selling animal meat products claimed to have never received an antibiotic (antibiotic-free, ABF; no antibiotics ever, NAE) has created a market for products that can provide the benefit of AGPs without using antibiotics that are used therapeutically in human medicine. The most important development in the search for credible alternatives to AGPs is the new understanding in both humans and veterinary animals that animals including humans are “superorganisms” that contain trillions of bacteria, with more than thousands of species, and that the gastrointestinal tract is an intelligent sensory organ that not only absorbs nutrients, but also communicates with the largest neuroendocrine system in the body. This new scientific knowledge in our understanding of the importance of the gut environment and barrier function in health should guide finding a future solution to develop novel products that can deliver the benefits of AGPs without causing an increase in the emergence of resistance. For example, when we consider using phytochemicals as antibiotic alternatives, we need to consider: (1) dose for immune versus bacteriostatic/cidal effect in target animals; (2) variations in active compound in plants and plant-derived products; (3) unexplored concurrent effects of phytochemicals (antiviral and antineoplastic); (4) target organs/tissues affected by phytochemicals; (5) safety of phytochemical residues in humans; and (6) the long-term effect of using phytochemicals in animals on developing resistance. Since using phytochemicals as antibiotic alternatives in agricultural animals is a relatively new field of research, regulation of these products poses a challenge. There is a timely need to provide increased public funding for mechanistic research for phytochemicals that include standard measurements to define the efficacy in a consistent manner across several regulatory pathways, to prevent false claims and yet have flexibility in the approval process for proof of efficacy or safety for commercialization. Owing to the rise in consumer demand for livestock products from ABF production systems, scientists, regulatory agencies and commercial partners need to work together to develop effective antibiotic alternatives to improve performance and maintain optimal health of food animals. Using optimal combinations of various alternatives coupled with good management and husbandry practices will be the key to maximizing performance and maintaining animal productivity, while we move forward with the ultimate goal of reducing antibiotic use in the animal industry. Further research is needed regarding understanding their mechanism of action, identifying means to standardize the effects, improving delivery methods (e.g. microencapsulation) for site-targeted delivery, and increasing their in vivo efficacy in farm settings.

## Abbreviations

ABF: antibiotic-free
AGPs: antibiotic growth promoters
DM: dry matter
EOs: essential oils
FDA: Food and Drug Administration
HMG-CoA: hydroxymethylglutaryl coenzyme A
IFAs: in-feed antibiotics
IFN: interferon
IL: interleukin
LPS: lipopolysaccharide
NAE: no antibiotics ever
NE: necrotic enteritis
NO: nitric oxide
OIE: World Organization for Animal Health
OUT: operational taxonomic units
PTS: propyl thiosulfinate
PTSO: propyl thiosulfinate oxide
SCFA: short-chain fatty acid
SFBs: segmented filamentous bacteria
TNFSF15: TNF superfamily member 15
VFAs: volatile fatty acids
VFD: Veterinary Feed Directive
